# Exploring the experience of community hospital-led home-based cardiac rehabilitation health management in patients with coronary heart disease: a qualitative focus group study

**DOI:** 10.3389/fcvm.2025.1748801

**Published:** 2026-01-12

**Authors:** Lin Wu, Yanyan Song, Ruolan Zhang, Shining Lou, Quanwei Di, Qisong Shi, Lishu Peng, Xian Chang, Ning Liu, Haiming Li, Yan Wang

**Affiliations:** 1 School of Nursing, Hebei University, Baoding, Hebei, China; 2 Baoding Hospital Affiliated to Beijing Children’s Hospital, Capital Medical University, Baoding, Hebei, China; 3 Work’s Hospital of China Lucky Group Corporation, Baoding, Hebei, China; 4 The Second Hospital of Baoding, Baoding, Hebei, China; 5 Affiliated Hospital of Hebei University, Baoding, Hebei, China

**Keywords:** community hospitals, coronary heart disease, experience, home-based cardiac rehabilitation, qualitative focus group study

## Abstract

**Objective:**

To explore experiences and perspectives on community hospital-led home-based cardiac rehabilitation for coronary heart disease patients.

**Methods:**

Semi-structured, face-to-face focus group interviews were conducted at a community hospital in Baoding, China, from December 2024 to October 2025. The interview guide was developed on the study objectives and rigorous internal deliberations within the research team. Data analysis was performed using Colaizzi's 7-step method.

**Results:**

Three focus group interviews were conducted involving 15 coronary heart disease patients. Data analysis resulted in the identification of five themes and 15 subthemes. The five themes are presented as follows: motivations for engaging in cardiac rehabilitation and cognitive shifts; positive experiences and perceived benefits of cardiac rehabilitation; challenges in self-managing during home-based cardiac rehabilitation; expectations and suggestions for cardiac rehabilitation services; and meaning-making in disease management and life course.

**Conclusion:**

Cardiac rehabilitation experiences for coronary heart disease patients are a dynamic process involving physiological, psychological, and behavioral adaptations, which present multidimensional challenges. Developing a patient-centered, community-based support system requires integrating several key elements: culturally congruent strategies, multidisciplinary collaboration, personalized protocols, continuous monitoring, and robust psychosocial support. This holistic approach empowers patients to transition from a passive state of illness to active health stewardship, thereby achieving sustainable long-term outcomes.

## 1 Introduction

Coronary heart disease (CHD) is one of the most prevalent cardiovascular diseases worldwide, having a substantial impact on patients' quality of life and survival outcomes (1). Approximately 220 million individuals worldwide are affected by CHD, resulting in an estimated 9 million annual deaths, which accounts for 16% of global mortality (2). In China, with the acceleration of the aging population, the incidence and mortality rates of CHD are projected to continue rising, posing a significant threat to public health and imposing a substantial socioeconomic burden (3).

Cardiac rehabilitation (CR) is a cornerstone of CHD management (4). Following a comprehensive medical assessment, CR delivers continuous, integrated care through structured prescriptions targeting modifiable risk factors to optimize secondary prevention (5). As a core strategy, CR improves patient outcomes, slows atherosclerosis progression, and significantly reduces the risks of disease recurrence, rehospitalization, and all-cause mortality while enhancing quality of life (6–8). Despite these established benefits, global CR utilization remains suboptimal at 54.7%, with substantial disparities in access across regions (9). Traditional hospital-based CR programs, despite being highly specialized, are plagued by low participation, high dropout rates, and increased healthcare burdens. These issues stem from patient-level barriers, such as transportation costs and time constraints (10), as well as systemic challenges like limited funding and program availability (11, 12). In response, major professional societies, including the American Association of Cardiovascular and Pulmonary Rehabilitation, the American Heart Association, and the American College of Cardiology, have jointly endorsed home- and community-based CR as a viable alternative model (13). A critical challenge in this model, however, is that once patients return to the community, healthcare providers struggle to monitor their rehabilitation progress in real-time and obtain timely feedback. This often leads to a misalignment between rehabilitation programs and patients' actual needs, ultimately impairing self-management capabilities and long-term adherence (14).

Home-based cardiac rehabilitation (HBCR) is an established model that primarily utilizes the home environment to deliver long-term, and in some cases lifelong, rehabilitation guidance, offering patients a more economical and convenient recovery model (13). Internationally, there is a clear trend shifting from traditional center-based rehabilitation toward home, community, and remote models (15). CR is frequently implemented in community-based healthcare settings outside hospitals, including community hospitals, intermediate care facilities, and clinics (16–18). Research evidence indicates that personalized CR utilizing telehealth technologies and wearable devices can deliver comparable benefits to center-based CR in improving cardiovascular function and outcomes (15), while offering significant advantages in terms of accessibility and patient engagement (19, 20). Although CR in China was initiated relatively late, it has since developed rapidly. To address challenges such as a substantial patient population and uneven distribution of medical resources, an integrated hospital-community-home model has been established as a cornerstone strategy (21). This model aligns with the national health policy to decentralize the management of chronic diseases like CHD to the community level. It leverages the strengths of community healthcare providers to promote the sustainable development of CR. This approach offers a dual benefit: it alleviates well-documented challenges within the Chinese healthcare system, such as difficulty in securing appointments, high costs, and limited resources at tertiary hospitals, while it simultaneously channels enhanced resources and guidance to community hospitals (22). Preliminary studies confirm that such models improve patient outcomes by facilitating access to rehabilitation services within local communities, which significantly enhances both the accessibility of and adherence to CR (23, 24). The full realization of these benefits, however, is contingent upon patients' sustained participation and effective self-management in the home setting. Therefore, empowering patients to achieve self-management mastery constitutes the ultimate goal of CR (25).

However, this foundational premise remains underexplored, and patient-centered research delving into the authentic experiences of HBCR is still lacking. Although qualitative inquiry into CR has gained increasing global attention, most recent studies focus on virtual or remote rehabilitation experiences (26, 27). This body of work often overlooks the perceptions of patients navigating the behavioral restructuring phase of CR in resource-constrained settings. Furthermore, many studies approach HBCR from the perspectives of healthcare providers and administrators, neglecting patients' actual experiences, adaptation processes, and ongoing challenges within the resource-limited setting of everyday home environments (28, 29). Furthermore, cultural backgrounds, healthcare services, CR models, and medical guidelines vary across countries. Previous studies indicate that China's unique family culture and social context also influence patients' CR plans (30). The inner journey of how patients comprehend, adapt to, and adhere to rehabilitation plans, along with the emotional and social challenges they face, remains underexplored. This gap seriously hinders the development and implementation of personalized, actionable cardiac rehabilitation programs. Therefore, this study employs focus group interviews to explore patients' authentic experiences of CR health management within home environments. This approach aims to achieve a deeper and more comprehensive understanding of patients' perceptions, needs, and challenges regarding community hospital-led HBCR. The findings will provide evidence-based insights to optimize self-management strategies for CHD patients in home settings and develop more personalized CR programs.

## 2 Materials and methods

### 2.1 Design

This study employed a descriptive phenomenological design. Data on the community hospital-led HBCR experiences of patients with CHD were collected via focus group interviews, transcribed verbatim, and analyzed using Colaizzi's seven-step phenomenological analysis method (31). This process involved extracting meaningful statements relevant to the interview guide, coding recurring concepts, and synthesizing these into overarching themes. The study adhered to the Consolidated Criteria for Reporting Qualitative Research (COREQ) guidelines (32).

### 2.2 Study setting and recruitment

The study was carried out from December 2024 to October 2025 at a multipurpose conference room of a community hospital in Baoding, China. To ensure sample diversity and representativeness, purposive sampling with maximum variation was employed, capturing heterogeneity across gender, age, educational attainment, marital status, occupation, family history, and residential type. After obtaining cooperation from community staff, researchers conducted face-to-face communication with patients undergoing CR health management at the community CR clinic. They provided detailed explanations of the study objectives and invited participants to join the study. Informed consent was obtained from interested participants, and interview times and locations were scheduled. Sample size was determined by the principle of data saturation, defined as the point where no new thematic codes emerged in the final focus group interview, and existing codes showed repetition. Ultimately, 18 patients were invited to participate, and three declined due to a lack of interest.

### 2.3 Inclusion and exclusion criteria

Inclusion criteria: (1) Diagnosis of CHD; (2) Age≥18 years; (3) Minimum 3-month participation in a community hospital-led HBCR program; (4) Adequate communication and cognitive abilities; (5) Provision of voluntary informed consent.

Exclusion criteria: (1) Presence of malignant tumors or severe complications; (2) Physical activity limitations due to other diseases or contraindications to exercise; (3) Participation in other trials or rehabilitation programs within the past 3 months.

### 2.4 Data collection

All semi-structured focus group interviews in this study were facilitated by WL, and SYY acted as the assistant facilitator. The facilitator guided the interview process, while the assistant documented the proceedings. The facilitator (WL) is a graduate student specializing in CR research, holds a registered nurse license, and has clinical experience caring for CHD patients. The assistant facilitator (SYY) is also a graduate student specializing in CR research. The research team also included a professor who completed mentor training accredited by the Cardiovascular Disease Prevention and Rehabilitation Professional Committee of the China Heart Alliance and holds national certification as a cardiopulmonary rehabilitation therapy and nursing mentor. All researchers were female, had completed CR-related training, obtained completion certificates, and received systematic training in qualitative research methodology. Before the interviews, a trusting relationship was established between the researchers and the study participants. However, the participants' understanding of the researchers was limited to the information required for their study participation. The interview guide was developed based on the study objectives and rigorous internal deliberations within the research team. It was refined after three individual pre-interviews with CHD patients. Data obtained from these pre-interviews were excluded from the final analysis. During the pre-interviews, all study participants reported feeling anxious when interacting with healthcare providers, sometimes uncertain about how to articulate their genuine feelings, and expressed a preference for gathering with other patients to discuss relevant topics. Subsequent to the pre-interviews, the research team conducted content analysis to finalize the interview format and guide (Table 1), which was determined to be focus group interviews. Focus group discussions were conducted in a multipurpose meeting room at the participating community hospital. All participants and researchers were present throughout each session, and deliberate efforts were made to cultivate an open atmosphere that encouraged participants to share personal experiences and speak freely. Following written informed consent from participants, each entire session was fully audio-recorded and videotaped, and research team members concurrently documented field notes to capture nonverbal cues and key thematic points. A participant validation mechanism was implemented, involving regular checks for understanding and a summary of responses at the conclusion of each session. Data saturation was reached in the third focus group, and no participant was interviewed more than once. In total, 15 participants were interviewed across the three focus groups, with each session lasting 80–95 min.

**Table 1:** Semistructured interview guide.

Question number	Semistructured interview questions
1	When you first initiated cardiac rehabilitation, what were your initial expectations? Following your participation in the home-based cardiac rehabilitation program, what has been your overall experience with the intervention?
2	How does the current reality compare to your initial expectations?
3	Based on your experience during this period, what do you perceive “cardiac rehabilitation” primarily refers to?
4	How do you typically incorporate rehabilitation activities into your day?
5	During your exercise routine, what situations make it difficult for you to stick with it? What situations motivate you to keep going?
6	What are your thoughts on consistently following the rehabilitation plan recommended by medical professionals over the long term?
7	What do your family or friends think about your commitment to cardiac rehabilitation?
8	What specific gaps or limitations do you perceive in healthcare providers' home-based cardiac rehabilitation management for community-dwelling coronary heart disease patients? To better deliver home-based CR services to community-dwelling coronary heart disease patients, what specific improvements would you suggest for healthcare providers?

### 2.5 Data analyses

Within 24 h of each interview session, two researchers independently transcribed the audio recordings and video verbatim into textual data, documenting the study participants' nonverbal cues during the interactions. Both researchers conducted multiple readings of the transcripts to achieve a comprehensive understanding of the data and ensure accuracy. Following anonymization of all identifying information, the textual data files were imported into NVivo 12.0 software for data management and analysis. Data analysis was conducted using Colaizzi's seven-step analysis method for the Chinese-language dataset (31), with strict adherence to all seven steps throughout the analytical process. (1) Familiarization: Both researchers achieved a holistic understanding of CHD patients' participation in community-led home-based CR health management through careful and repeated reading of the transcripts. (2) Extraction of significant statements: Identification and extraction of important, meaningful statements related to CHD patients' subjective willingness and psychological experiences regarding independent home-based CR health management. (3) Derivation of meaning units: Categorization and coding of similar viewpoints into discrete meaning units. (4) Clustering of themes: Grouping of similar codes into more abstract, overarching themes. (5) Development of comprehensive descriptions: Construction of a thorough description of the phenomenon based on the identified overarching themes. (6) Development of a basic structure: Organization of the expressed meanings into themes and subthemes to reflect the essence of the participants' experiences. (7) Member checking: Researchers WL and ZRL integrated all themes and descriptions, then returned the resulting thematic structure to the participants to verify whether their authentic experiences and feelings were captured, thereby ensuring the trustworthiness of the findings. The same data were independently analyzed by two researchers. When disagreements arose, a third researcher reviewed the original materials and facilitated discussions until consensus was reached. Through collective deliberation, findings were compared and synthesized to establish the final thematic framework. Example of partial analysis: “Too tired after returning home, and without supervision, I don't exercise.” This statement was transformed into the meaning unit “Challenges of sustaining rehabilitation Exercise.” Further, this meaning unit was grouped under the theme “Multiple challenges in long-term health behavior adherence.”

### 2.6 Ethical considerations

Ethical approval was obtained from the Research Ethics Review Committee of Hebei University Hospital (No. HDFYLL-KY-2023-130).

### 2.7 Rigor and reflexivity

To ensure methodological rigor, this study adhered to Guba and Lincoln's framework of trustworthiness (33). Credibility: The interview guide was developed on the study objectives and internal team discussions. Maximum variation sampling was employed to ensure sample diversity. Researchers possessed extensive experience in community and CR work, establishing strong rapport with participants to guarantee data authenticity and depth. Reliability: All researchers received systematic qualitative research training, possessing professional research skills and rigorous academic integrity. Experts familiar with Colaizzi's method were invited to analyze data, reaching consensus through discussion to enhance reliability. Confirmability: During interviews, comprehensive techniques probing, paraphrasing, summarizing, clarifying were repeatedly applied to obtain thorough, accurate information. Researchers minimized coding subjective bias by quoting participants' original statements and seeking iterative confirmation. Transferability: Comprehensive descriptions of the study's research design and analytical processes were provided to enhance findings transferability to similar contexts. Reflexivity (34): Before the study, researchers reflected on personal values and professional backgrounds' potential influence. Throughout data collection and analysis, reflexivity was maintained via field notes and analytical memos to further minimize subjective bias.

## 3 Results

A total of 3 focus groups were conducted, each comprising 4–6 participants, for a total of 15 participants. The mean participant age was 65.5 years (range 42–79). Participant characteristics are detailed in Table 2. During data analysis, the final analysis yielded 165 codes distributed across 15 subthemes within 5 main themes (Table 3).

**Table 2:** Characteristics of participants.

Participant ID	Gender	Age (years)	Highest educational level	Occupation	Medical Insurance	Disease course (years)	Complications (types)	Family history	Residential type
P1	Male	75	High school	Retired	Employee medical insurance	24	2	None	Living with spouse
P2	Female	74	High school	Retired	Employee medical insurance	2	1	None	Living with spouse
P3	Female	66	Middle school	Retired	Employee medical insurance	3	2	None	Living with adult children
P4	Female	62	High school	Retired	Employee medical insurance	2	0	None	Living with adult children
P5	Male	68	High school	Worker	Employee medical insurance	30	0	None	Living with adult children
P6	Male	79	Associate Degree	Retired	Employee medical insurance	1	0	None	Living with spouse
P7	Male	77	High school	Retired	Employee medical insurance	3	0	None	Living with adult children
P8	Female	67	Middle school	Retired	Employee medical insurance	5	1	None	Living with adult children
P9	Female	69	Middle school	Retired	Employee medical insurance	2	1	None	Living with adult children
P10	Male	60	Master	Doctor	Employee medical insurance	2	2	None	Living with spouse
P11	Female	61	Undergraduate	Retired	Employee medical insurance	1	2	None	Living with spouse
P12	Female	69	High school	Worker	Rural Resident Medical Insurance	1	2	Yes	Living with spouse
P13	Male	56	High school	Farmer	Rural Resident Medical Insurance	3	1	Yes	Living with adult children
P14	Female	57	Middle school	Farmer	Rural Resident Medical Insurance	1	1	None	Living with spouse
P15	Male	42	Associate Degree	Worker	Rural Resident Medical Insurance	2	0	Yes	Living with spouse

**Table 3:** Five themes and fifteen subthemes.

Theme	Subthemes
1. Motivations for engaging in CR and cognitive shifts	Diversity of participation motivation
	The process of cognitive transformation
2. Positive experiences and perceived Benefits of CR	Improved physiological function
	Improved mental state
	Improved health behavior
3. Challenges in self-managing during HBCR	Limited knowledge internalization and execution capabilities
	Multiple challenges in long-term health behavior adherence
	Insufficient self-determination capability during sudden anomalies
	The objective benefits and challenges of mobile health wearables coexist.
4. Expectations and suggestions for CR services	Deepening and expanding health education content
	Optimization of rehabilitation guidance formats and the need for individualized adaptation of techniques
	The need to establish long-term doctor-patient partnerships
5. Meaning-making in disease management and life course.	Revolutionizing health concepts
	Role transformation: from passive patient to proactive health manager
	A positive mindset for living with illness

### 3.1 Theme 1: motivations for engaging in CR and cognitive shifts

#### 3.1.1 Diversity of participation motivation

Most patients reported that their initial motivation for participating in CR came from the belief that it would benefit their physical health and support recovery. P11 stated, “I simply believe cardiac rehab is good for my body and helps speed up recovery.” Other key factors influencing their decision to participate included the need for scientific guidance, easy access to medical resources, and a strong desire to maintain independence to lighten their children's burden. P2 explained, “As we grow older, illnesses are inevitable. They can't be avoided or reversed. But we don't want to rely on our children; they're all quite busy. If we have to ask them for care, we truly want to manage our own health well. That's also a way to support them.” This directly reflects patients' tendency to use rehabilitation to maintain independence, reducing their reliance on their children's care. P1 noted, “Many people know exercise benefits their condition, but they're not sure how to do it effectively. Your program needs to be based on solid science.” This reflects a clear demand for scientific validity in cardiac rehabilitation and stands out as a key motivator for participation. P7 added, “Another reason is that this hospital has a good reputation and is conveniently located, so we decided to come here.” This underscores the practical factors shaping patients' choice of CR programs.

#### 3.1.2 The process of cognitive transformation

Most patients indicated that they were experiencing profound shifts in their thinking during recovery. They move from initial skepticism and fear to acceptance and trust after seeing results for themselves, eventually embracing proactive self-management and a more positive outlook on life. P5 stated, “A classmate told me, ‘At your age, you shouldn't join this. You'll just end up being their guinea pig.’ But I said I'd already been participating for a while. They aren't after anything from me, and my health has actually improved. I truly believe this cardiac rehabilitation program is incredibly beneficial.” This statement highlights the initial skepticism patients faced from their peers and their own doubts. After participating, their perspective on CR changed. P8 noted, “At first, I didn't understand the point of it all. Every day felt like just checking off tasks, and it seemed pretty tedious. But now I can feel the benefits myself. I even told a friend to come here for rehabilitation, too.” It can be seen that shift from initial indifference to recognizing the tangible benefits of the program. P2 explained, “Before, we just did whatever the doctors told us without knowing why. Now, with your guidance, we understand how to manage our own health and make informed choices.” This illustrates the shift from passively following medical advice to actively managing one's health. P9 added, “Right after getting sick, I couldn't accept it and didn't want to go out. But through rehab, I'm getting better and better. You can still live well even with an illness.” This reflects a shift from psychological denial and withdrawal to acceptance and re-engagement with life.

### 3.2 Theme 2: positive experiences and perceived benefits of CR

#### 3.2.1 Improved physiological function

Several patients reported that regular participation in rehabilitation training has significantly boosted their physical strength and stamina. Cardiopulmonary discomfort symptoms have either lessened noticeably or disappeared entirely, leading to marked improvements in their overall functional capacity and quality of life. P9 conveyed, “My energy is much better now. I live on the 6th floor and climb the stairs twice a day. I can carry groceries like meat, pork knuckles and vegetables all the way up to the 6th floor with both hands, no problem.” P8 mentioned, “Before, I couldn't walk very far outside. Walking would make my heart feel uncomfortable, like it was being strained. That feeling's gone now.” These statements highlight the positive impact of CR on patients' daily functioning and well-being through tangible physiological benefits.

#### 3.2.2 Improved mental state

Some patients reported that participating in rehabilitation not only delivered positive emotional benefits but also significantly eased the fear and anxiety brought on by their illnesses. P12 expressed, “I have other health issues too, but exercising has really lifted my mood.” This embodies the emotional uplift and improved mental state resulting from CR exercise. P9 explained, “Back then, I was too scared to ride a bike. I worried someone might hit me. I was just so timid. But going through rehabilitation here has helped me understand my body better. Now when I ride, I don't feel afraid or anxious anymore.” This shows the reduction of anxiety and increased confidence in daily activities due to the knowledge and support gained through rehabilitation.

#### 3.2.3 Improved health behavior

Patients reported that rehabilitation guidance has transformed their health-related behaviors in meaningful ways. These changes aren't limited to specific actions like actively controlling their diet. They also involve creatively fitting regular exercise into the gaps of their daily routines, making health management a normal part of life. P4 shared, “Thanks to your advice, I've cut way back on sweets. I barely exercised before, but now, when I play mahjong, I set an alarm to get up and move around every hour or two. Sometimes I even exercise while watching TV.” This quote emphasizes how rehabilitation guidance has enabled patients to creatively incorporate exercise into everyday activities. P8 expressed the same view, “I always thought exercise meant going to the gym or some specific place, but you showed me I can use breaks while cooking to get moving.” This highlights the shift in patients' understanding of exercise, making it a seamless part of daily life.

### 3.3 Theme 3: challenges in self-managing during HBCR

#### 3.3.1 Limited knowledge internalization and execution capabilities

Most patients reported that after receiving short-term rehabilitation guidance at cardiac rehab clinics, they struggled to apply these principles consistently and correctly in their daily lives at home. P6 recounted, “I use a wristband to monitor my heart rate when I exercise. You set my target heart rate above 80, but I just can't keep it there. Even a little exertion makes my heart rate jump over 100. Once I'm back home, I have no idea how to maintain that target heart rate during my workouts.” This directly reflects the challenge of applying specific exercise guidance to the home environment. P3 revealed, “I haven't really stuck to the diet recommendations. When I get home, I forget what I should and shouldn't eat. If my appetite's good, I tend to eat more; if it's poor, I eat less.” Practices reflecting patients' inadequate mastery of health education knowledge impede healthy dietary behaviors. P7 added, “I also struggle to judge exercise intensity accurately. I know I should sweat a little after working out, but I've forgotten the other things you mentioned back then.” This reflects the fragmented and limited nature of patients' knowledge retention, hindering the scientific execution of rehabilitation plans during home self-management.

#### 3.3.2 Multiple challenges in long-term health behavior adherence

Numerous patients described struggling to stay motivated to overcome their own laziness when at home, where there's no external supervision. They also have little resistance to unhealthy habits. At the same time, shaped by traditional Chinese social norms, their deep-rooted social habits are hard to change. This makes it difficult to sustain healthy behaviors. P10 explained, “I drive a big truck and work from dawn till dusk. There are mandatory breaks scheduled, but there's always some small task popping up. Without anyone keeping an eye on me, I never think to move around. When I get home, I'm too tired to exercise. I just eat and sleep. I only feel like working out on days when I'm not as tired.” Work demands and personal inertia are important factors affecting patients' long-term healthy behaviors. P4 admitted, “I haven't stuck to the smoking cessation plan. My mahjong buddies always ask me to join them, and once I'm there, I end up smoking. We've been playing together for years, so it's hard to say no.” This highlights the difficulties patients face in sustaining healthy behaviors due to entrenched social habits and interpersonal relationships.

#### 3.3.3 Insufficient self-determination capability during sudden anomalies

In HBCR without timely professional guidance, some patients report feeling unsure how to properly respond to changes in their physical condition. They lack clear, scientifically sound criteria to guide their self-management decisions. P7 recounted, “Sometimes my heart feels uncomfortable, but I don't know how to deal with it. I figure it might go away in a day or two, and since it doesn't affect my exercise, I just brush it off.” Patients lack clear strategies for sudden discomfort, thereby adopting a passive approach to health issues. P8 expressed a similar sentiment, “I sometimes feel foggy-headed, but I don't know how to address it. I just ignore it and keep taking my blood pressure medication as usual.” P3 admitted, “At first, exercising felt great when my back didn't hurt. But lately, with the back pain, I'm not sure if I should keep going or stop. I never checked with a doctor about it, so I stopped exercising on my own. I haven't worked out lately, and now I feel a bit weak.” Directly reflects the uncertainty in decision-making when confronting physical discomfort.

#### 3.3.4 The objective benefits and challenges of mobile health wearables coexist

##### 3.3.4.1 Technology empowerment: real-time feedback for greater sense of security

Many patients noted that wearable devices let them intuitively track key metrics like heart rate and blood oxygen levels. This not only provides objective guidance for adjusting exercise intensity but also greatly boosts their sense of security and confidence when exercising at home. P9 recounted, “With this fitness tracker, I feel much more at ease when working out. It shows my target heart rate range and the maximum value I shouldn't go over. When I see it exceeds that number, I immediately slow down my activity.” P8 elaborated, “Before, when I exercised outdoors, I never knew what intensity was right. The biggest challenge was judging how hard to push myself. But after my cardiac rehabilitation doctor advised me on my target exercise heart rate, I stuck to those guidelines. When the exercise timer goes off, I know I've done enough and can stop.” Both illustrate that patients regard intuitive data feedback from wearable devices as a positive value for ensuring safe exercise.

##### 3.3.4.2 Technological burden: data anxiety and usage burden

However, while wearable devices offer convenient health monitoring, they also create unique technological barriers for older adults. These stem from the complexity of interpreting data, operational inconveniences, and the psychological stress that follows. P7 said, “My sleep has become a big issue. I never meet the recommended standards. Since my fitness tracker syncs with my phone, I check my sleep data the second I wake up every morning. It keeps telling me I'm severely lacking deep sleep, and that's really distressing.” This highlights the psychological stress and data anxiety caused by constant monitoring and negative feedback. P12 explained, “I had surgery on this arm and never got used to wearing a wristband. Now I have to remember to put it on every day. It feels like such a hassle.” This underscores the operational inconvenience and additional burden of using wearable devices. P8 added, “My eyesight's getting worse. The text on this wristband is way too small. It's really hard for me to read.” This illustrates the physical challenges and usability issues that further complicate the use of wearable devices for older adults.

### 3.4 Theme 4: expectations and suggestions for CR services

#### 3.4.1 Deepening and expanding health education content

After CR, many patients expressed a need not only for personalized rehabilitation guidance but also for general health content that would encourage family involvement. They also noted a desire for continued access to health information from professional teams. This information should be easy to understand and regularly updated. P7 expressed a need for health education materials that are easy to understand and promote family involvement, stating, “I hope you can share more effective, easy-to-understand methods for medications, exercise, and diet. That way, my family and I can stick to these practices together. After all, you're the experts in this field.” P9 added, “As professionals who specialize in this area, I think you could develop new exercises or add more content. For example, how do newly developed medications help the heart, and what effects do they have? You could also share some fresh insights with us.” This indicates that patients are more inclined to seek new insights and updates on the disease and its treatment from professional teams.

#### 3.4.2 Optimization of rehabilitation guidance formats and the need for individualized adaptation of techniques

Regarding the feasibility and adherence of rehabilitation, many patients hoped for more intuitive, user-friendly formats for rehabilitation guidance. These could include easy-to-follow video tutorials and tech tools that fit different personal usage habits. They also wanted more diverse exercise options to address home practice challenges, such as trouble remembering movements, operational difficulties, and lack of persistence. P5 proposed, “I can't remember some of the movements when I get home. Could you make a video? Maybe add music to it, like square dancing videos.” Patients expressed the need for more intuitive and engaging rehabilitation guidance formats to enhance memory retention and improve adherence. P15 commented, “I always struggle to stick with cardiac rehab. I'd really prefer more varied exercise formats so I can switch things up and keep going consistently.” This directly reflects the need for diverse exercise options to maintain motivation and consistency.

#### 3.4.3 The need to establish long-term doctor-patient partnerships

Many patients noted that after working with the community CR team for over three months, they experienced the team's professionalism and sincere dedication. They hoped this connection would continue. P14 shared, “I truly believe cardiac rehab is essential for older adults today. Your team is dedicated, skilled, and competent. I can feel genuine care from all of you. We seniors may not be the youngest, but you still treat us with so much warmth. It really touches my heart. I hope you'll keep this work going.” This highlights the emotional impact of the team's care and the desire for ongoing support. P1 reflected, “At first, I actively sought you out at the clinic. Later, with your encouragement and guidance, it became clear how we should live a healthy life. It would be wonderful to stay connected with you.” This underscores the transformative role of the team and the need for sustained guidance. P6 exhibited the emotional impact of the team's care, expressing a desire for ongoing support, added, “Every time I visit the CR clinic, you greet me warmly and show genuine concern for every part of my health. Following your advice has been so beneficial. Please keep this rehabilitation work going.” This reinforces the positive impact of the team's warm and genuine care on patients' health outcomes and their wish for sustained support.

### 3.5 Theme 5: meaning-making in disease management and life course

#### 3.5.1 Revolutionizing health concepts

As attitudes toward rehabilitation evolve, many patients have shifted their health mindset from passive disease treatment to proactive prevention and self-management. They now see maintaining physical health as both a key asset for improving quality of life in later years and a personal responsibility. P11 commented, “As a doctor with other chronic conditions, I know cardiac rehabilitation doesn't just benefit the heart. It also helps manage hypertension, diabetes, and other illnesses. That's why I hope more people with chronic diseases will join cardiac rehabilitation programs.” This Illustrates the holistic view of health, recognizing that cardiac rehabilitation can benefit multiple chronic conditions and improve overall health. P7 added, “Building up our physical fitness now is like accumulating capital. It directly boosts our quality of life in our later years.”

#### 3.5.2 Role transformation: from passive patient to proactive health manager

##### 3.5.2.1 Regain a sense of health autonomy

Many patients reported that by participating in CR, they have gradually mastered scientific self-management skills. They can proactively adjust rehabilitation plans, such as diet and exercise, to match their personal rhythms. This shift allows them to move from passive treatment to active health management. P2 recalled, “I'd never heard of cardiac rehabilitation before. It was a complete mystery. But over these past three months, with your scientific guidance, we've kept a small notebook to track what we eat and our blood sugar levels. Gradually, we figured out which foods work for us and which don't. This hands-on approach is much better than just getting instructions from the doctor.” This indicates enhanced patient autonomy in health behaviors through self-regulation skills. P8 added, “You suggested using cooking time for exercise, and that worked really well for me. Now I can fully arrange my schedule based on my own availability.” This reflects a shift from passive compliance to active self-management in health care.

##### 3.5.2.2 Rebuild self-efficacy

The noticeable improvements in physical fitness from CR have translated into strong psychological momentum. This has restored their confidence in daily life and nurtured a positive self-image. P8 said, “After over three months, my stamina has improved a lot. Before, whenever I went out to do things, my heart would feel tight. Now that feeling's gone completely. I also feel confident enough to take on so many more activities.” P1 exclaimed, “I feel amazing now. I've stuck to the exercise routine every day since we started. I can climb stairs, go grocery shopping, anything. Everything else feels great too (Thumbs up).” Both reflect the positive psychological changes and enhanced sense of well-being in daily life following improvements in physical fitness through cardiac rehabilitation.

##### 3.5.2.3 Persistent anxiety during the transition process

Even with participation in structured rehabilitation management, lingering symptoms or unmet improvement expectations can still trigger worries and anxiety about recovery outcomes and long-term health management. P7 lamented, “I think my sleep issues are different from other people's. Right now, my sleep quality is really poor. I've tried all kinds of things for over three months. That includes the sleep-promoting exercises you taught me, ones I found online, and other techniques. But there hasn't been much improvement.” P8 confessed, “I've been taking my blood pressure medication exactly as the doctor prescribed. Taking it just once in the morning wasn't enough to lower my blood pressure. We adjusted the dosage once, adding a second dose at night. It's been two or three months now, and my blood pressure is still not where it should be. It's really frustrating.” Both separately illustrate anxiety and frustration arising from unimproved sleep issues and unmet blood pressure control expectations.

#### 3.5.3 A positive mindset for living with illness

Some patients report having learned to coexist peacefully with their illness. By clearly recognizing their limitations, proactively adjusting their lifestyle, and integrating medical advice with personal insights, they have achieved an active life within safe boundaries. P4 noted, When hiking, I definitely avoid climbing too high and take the cable car instead. Walking a bit is fine, but I understand my physical condition and pace myself accordingly. It exemplifies a practical approach to maintaining an active lifestyle within personal health limits. P9 stated, Since I'm already sick, I just go with the flow. I take my medications as prescribed and follow the usual precautions. Patients reflects an adaptive attitude toward illness, highlighting the importance of adherence and self-awareness. P8 expressed the same opinion, While I use a fitness tracker to monitor my heart rate during exercise, I also rely on how I feel. If walking too fast makes me uncomfortable, I naturally slow down.

## 4 Discussion

This study conducts an in-depth analysis of the home-based CR experience of CHD patients under the leadership of community hospitals. It reveals that this process is not merely the implementation of clinical protocols but a complex, dynamic process centered on the disease and CR, encompassing psychological adaptation, behavioral restructuring, and social interaction. This study found that community hospitals not only promote patients' cognitive and behavioral changes through professional guidance during the early stages of rehabilitation, but also serve as the core support for patients' psychological well-being, technology use, and health decision-making in long-term management. Against the backdrop of limited CR resources, inadequate self-management, and low adherence among patients, the findings of this study not only provide precise directions for interventions aimed at developing self-management strategies for patients with CHD but also offer critical scientific evidence for establishing and optimizing community hospital-led CR management models that align with patient needs and ensure sustainability. The challenges patients face when using wearable devices highlight the need for user-friendly tools. Their need for ongoing support underscores the importance of robust support networks. Limited cardiac rehabilitation resources highlight the crucial role of community hospitals in integrated care. Future model optimization should further strengthen the pivotal role of community hospitals as hubs for resource integration, technology adaptation, and continuity of care support.

Our findings indicate that patients' perceptions of HBCR evolve from initial skepticism to trust in the program. This cognitive shift is driven by multidimensional motivations across personal, familial, and societal levels. Consistent with the Capability, Opportunity, Motivation, and Behavior (COM-B) model framework (35), this cognitive transformation, together with emotional responses, further strengthens patients' motivation for CR. The transition in patients' self-identity from passive care recipients to active health managers supports the core tenets of Self-Determination Theory. This theory emphasizes that satisfying individuals' needs for autonomy, competence, and relatedness is critical to fostering intrinsic motivation and sustaining long-term healthy behaviors (36). Specifically, patients regain a sense of self-control by monitoring their health indicators independently and integrating rehabilitation activities into daily life, which reflects enhanced autonomy. Meanwhile, the reconstruction of self-efficacy, driven by improved physical function and restored confidence, demonstrates the development of a sense of competence. Other studies have also confirmed that promoting patients' active health management can effectively guide positive changes in health behaviors (37). Furthermore, unlike some studies focusing solely on the positive dimensions of the transition, this research also uncovers psychological vulnerability during the role-shifting process. Some patients experienced persistent anxiety after investing effort, as residual symptoms such as sleep disturbances and inadequate blood pressure control did not improve as expected. This finding validates that transitional anxiety significantly impairs patients' self-management capabilities. It further reveals that the shift from passive dependency to active management is not a linear process (38) but a dynamic journey marked by psychological tension. Thus, “transition anxiety” should be recognized as a clinical indicator requiring routine assessment and intervention, rather than merely a personal emotional concern. Compared to studies centered primarily on positive transition factors, this research's value lies in identifying this critical determinant of transition quality. It emphasizes the urgent need to integrate sustained psychological support into HBCR management, helping patients establish realistic expectations and enhance their resilience to setbacks. This is pivotal for preventing loss of confidence and discontinuation of rehabilitation plans. In clinical practice, therefore, efforts should be made to reinforce patients' roles as active managers by empowering them with rehabilitation choices and stepwise skill-building. Simultaneously, interventions targeting transition anxiety must be incorporated into core workflows. Through expectation management, dynamic screening, and targeted psychological support, clinicians can effectively mitigate patients' psychological vulnerability and ensure the sustainable progression of rehabilitation plans.

While the home environment offers a convenient setting for CR, it also poses unique management challenges distinct from those in controlled clinical settings. In the absence of continuous professional guidance at home, patients often misinterpret or misapply complex medical advice. This, combined with fragmented outpatient CR education (39), hinders patients from mastering practical skills such as heart rate monitoring and resistance band usage following their return home. It reflects a tendency among outpatient clinical staff to prioritize one-way knowledge dissemination, while neglecting the critical importance of patients' comprehension and acceptance levels (40). Unlike Knudsen's (41) findings that telerehabilitation improves health literacy and delivers superior outcomes, this study identifies deficiencies in patients' ability to address health-related changes in the home setting, where they continue to adopt passive, wait-and-see coping strategies (42). However, it is noteworthy that this study identified significant decision-making deficits even among patients receiving standardized management, underscoring the limitations of current rehabilitation programs in training emergency response skills. The research further reveals the cultural logic underlying patients' long-term adherence challenges, which stem not only from individual inertia but are also deeply rooted in social pressures shaped by “interpersonal relationships” and “face-saving” (43). Crucially, this study found that the influence of “face” has extended from social interactions to doctor-patient dynamics, manifesting as patients delaying necessary consultations due to a fear of “disturbing” healthcare providers. This discovery highlights more complex constraints within the power dynamics of the doctor-patient relationship (44). Therefore, in clinical practice, optimizing CR outpatient education models is essential. This involves shifting from one-way knowledge dissemination to interactive skill training, such as reinforcing proficiency in heart rate monitoring and resistance band usage through simulation exercises and feedback-driven correction, while incorporating emergency response drills for scenarios like sudden blood pressure spikes or exercise-induced discomfort. Additionally, establishing convenient home-based consultation channels can prevent consultation delays caused by patients' psychological burdens. Furthermore, CR nurses and physicians can integrate family support guidance into rehabilitation plans. Peer support groups can facilitate patients' exchange of experiences in coping with social pressures, thereby alleviating the constraints of cultural factors on long-term adherence.

This study further identifies that CHD patients receiving HBCR management have multidimensional, multilevel needs. Patients demonstrate a strong desire for knowledge empowerment, with their needs extending beyond individual disease management to encompass leadership in overall family health. This signifies a shift in their role from passive recipients of treatment to active health advocates, an observation consistent with prior research showing that involving family members in health education significantly improves patients' adherence to health behaviors (45). Furthermore, patients' urgent demand for standardized exercise instructional videos underscores current limitations in translating exercise guidance into practical application. Effective knowledge transfer requires tangible, actionable tools, and such audiovisual media not only standardize movements but also support the development of regular exercise habits (46). Most critically, patients express a strong preference for establishing long-term, collaborative partnerships with healthcare providers. Characterized by professionalism, empathy, and sustained engagement, these partnerships deliver essential psychological security for effective self-management (44). Therefore, clinical CR healthcare professionals should adopt a personalized framework centered on shared decision-making. This includes integrating family health into health education content and developing concrete exercise guidance tools. Simultaneously, they should foster collaborative physician-patient relationships through regular follow-ups and jointly established rehabilitation goals, providing both knowledge and psychological support for patient self-management. Additionally, leveraging the professional expertise of cardiologists, healthcare providers should strengthen collaborative mechanisms between specialized and primary care CR teams for public health education. Deepening cooperation with community resources will expand the coverage and accessibility of CR services, addressing the large-scale rehabilitation needs of the chronic disease population.

Mobile health wearables offer both objective benefits and challenges in CR. On one hand, by enabling real-time visualization of physiological data such as heart rate, these devices provide patients with objective safety assurance and directly address the exercise-related anxiety commonly experienced during rehabilitation (47). On the other hand, this study found that when patients become overly fixated on the data itself, the device may shift from an aid to a source of stress. Some patients experience anxiety from failing to meet target metrics or becoming distracted from the exercise experience by deliberately trying to maintain specific values (48). This finding significantly complements prior research, which has predominantly focused on the advantages of wearable devices (49–51). Our study not only highlights potential negative experiences and psychological burdens in real-world applications but also uncovers the inherent contradictions and latent risks of wearable devices in clinical practice. Therefore, in clinical practice, when promoting wearable devices to support CR, it is essential to move beyond merely teaching patients how to monitor their data. Instead, efforts should be made to equip patients with the ability to interpret different data points and to implement psychological adjustment interventions. This approach prevents the devices from evolving from tools for safety assurance into sources of psychological stress for patients.

Furthermore, our findings confirm that social support is deeply embedded in patients' rehabilitation motivation and behavioral maintenance, serving as an indispensable source of psychological belonging (30). Families provide patients with the strongest economic and emotional support, and healthcare providers should encourage family members to engage in communication with patients and offer emotional support. In community HBCR practice, therefore, social support is not merely an external supplement but a core element that stimulates patients' intrinsic motivation, sustains their sense of self-efficacy, and ultimately underpins long-term CR health management.

## 5 Strengths and limitations

The strengths of this study lie in its use of focus group interviews, where group interaction encourages participants to share authentic experiences and reveals the complex motivations behind individual behaviors through the exchange of perspectives. Additionally, selecting samples from different communities within a localized area facilitates the identification of variations in health perceptions within specific cultural contexts. However, this study also has certain limitations. First, the generalizability of qualitative findings is limited. Second, although the sample size was relatively small, thematic saturation was achieved, preventing potential information redundancy. Finally, all participating CHD patients resided with either children or spouses; interviews with CHD patients living alone might provide additional insight.

## 6 Conclusion

This study used semi-structured interviews to explore the real experiences of CHD patients participating in HBCR health management programs run by community hospitals. Most patients undergo a shift from passive patients to active health managers, but this process involves significant challenges in self-managed home CR. Practical hurdles emerge in areas like exercise intensity control, dietary adherence, and retention of rehabilitation techniques. Patients have diverse needs, including personalized guidance, ongoing motivation, and technical support. Additionally, mobile health tools such as fitness trackers offer objective benefits while also posing unique challenges. Clinical practice can be improved through three key approaches: tool innovation, support network development, and resource integration. These efforts will help transition community-based CR from short-term interventions to lifelong health promotion.

## Data Availability

The original contributions presented in the study are included in the article/Supplementary Material, further inquiries can be directed to the corresponding author/s.
